# Sociological Society

**Published:** 1904-05-21

**Authors:** George Valentine


*To the Editor of* The Hospital.

### SOCIOLOGICAL SOCIETY.

*To the Editor of* "The Hospital."

Sir,—Will you kindly say where particulars as to the working of the Sociological Society can be obtained.

Yours truly,

George Valentine.

[The required information may be obtained from the hon. secretary of the Sociological Society, 5 Old Queen Street, Westminster, S.W.—Ed. "The Hospital'."]

